# Health promotion initiatives at school related to overweight, insulin resistance, hypertension and dyslipidemia in adolescents: a cross-sectional study in Recife, Brazil

**DOI:** 10.1186/s12889-018-5121-6

**Published:** 2018-02-07

**Authors:** Myrtis Katille de Assunção Bezerra, Eduardo Freese de Carvalho, Juliana Souza Oliveira, Eduarda Ângela Pessoa Cesse, Pedro Israel Cabral de Lira, Jonathan Galvão Tenório Cavalcante, Vanessa Sá Leal

**Affiliations:** 1 Centro de Pesquisas Aggeu Magalhães, Fundação Oswaldo Cruz (Fiocruz): Instituto Aggeu Magalhães, Av Prof. Moraes Rego, s.n. Campus UFPE, Cidade Universitária, Recife, PE CEP: 50.740-465 Brazil; 2 Universidade Federal de Pernambuco, Recife, PE Brazil; 3 Universidade de Brasília, Brasília, DF Brazil

**Keywords:** School health, Obesity, Physical education and training, Food habits, Adolescents

## Abstract

**Background:**

The emergence of diseases such as dyslipidemia, systemic arterial hypertension, insulin resistance and metabolic syndrome in children and adolescents has brought about a change in the epidemiologic profile of the pediatric population. As action to promote health in the school environment is a useful tool for changing the pattern of health/disease in the young population, the present study aimed to identify schools that promote healthy eating and physical activity and to study the relationship between these practices and the prevalence of overweight, hypertension, insulin resistance and hypercholesterolemia in adolescents.

**Methods:**

A cross-sectional population-based study was conducted with 2400 adolescents aged from 12 to 17 years old and participating in the “Study of Cardiovascular Risk in Adolescents” (ERICA – Estudo de Riscos Cardiovasculares em Adolescente). The association between dependent (overweight, insulin resistance, hypertension and dyslipidemia) and independent variables (implementation of health promoting initiative in schools) was investigated using the chi-square test and prevalence ratio (PR) with a confidence index (CI) of 95%.

**Results:**

The unsatisfactory implementation of a “health promoting environment” (PR = 1.02; CI 95%: 1.0; 1.04) and “partnerships with the health sector” (PR = 1.03; CI 95%: 1.01; 1.05) were linked to a high prevalence of overweight in adolescents. Hypercholesterolemia was found to be higher in the schools with unsatisfactory implementation of “healthy eating and health on the scholar curriculum” (PR = 1.71; CI 95%: 1.22; 2.44) and those lacking a “healthy-eating promoting environment” (PR = 1.29; CI 95%: 1.10; 1.54). Schools with unsatisfactory implementation of a “health-eating promoting environment” (PR = 1.36; CI 95%: 1.04; 1.79) and those lacking “partnership with the health sector” (PR = 2.12; CI 95%: 1.38; 3.24) had more adolescents with insulin resistance. There was no association between hypertension and any other component studied.

**Conclusion:**

Schools which have implemented adequate health promotion in their curriculums showed a lower prevalence of overweight, insulin resistance and hypercholesterolemia in adolescents.

## Background

In Brazil, about 25% of adolescents are overweight [1]. Overweight and obesity are among the main risk factors for the development of Chronic Non-communicable Diseases, and are related to premature death and disability in adult life [2–4]. Studies have shown concerning proportions of dyslipidemia, systemic arterial hypertension, insulin resistance and metabolic syndrome among children and adolescents, which have caused changes in the epidemiological profile of the pediatric population [5–8].

This situation is related to the fact that they are growing in an obesogenic environment which encourages weight gain, obesity and comorbidities caused by the energy imbalance resulting from changes in food types, availability, accessibility and commercialization; as well as a decline in physical activity which may be related to lower accessibility to places for practicing sports/exercise, and greater time spent on sedentary leisure (television, computer, video games, cell phones and tablets) [4, 9].

In this sense, regulatory policies and actions are recognized as one of the more cost effective means of dealing with complex problems such as obesity [10]. A coordinated effort to control overweight/obesity and its comorbidities requires intersectoral attention, especially from the health and education sector, with the intention of community participation.

Schools represent an appropriate environment for interventions aimed at promoting healthy lifestyles, since their physical, social and educational structure facilitates joint actions [11, 12]. Interventions that use an approach of health-promoting schools are able to reduce body mass index (BMI), increase activity levels and physical fitness, and improve fruit and vegetable intake by the students [12].

Many Brazilian schools are barely able to develop health promotion initiatives [13] for reason of lack of adequate environments and equipment for physical activity, as well as barriers to delivering healthy snacks and a lack of partnerships with the health sector and the community, and this may be harmful to the nutritional status of adolescents [12, 14, 15].

Considering that healthy promoting actions in the school environment represent a major challenge to public and private sectors management, yet also represent a great possibility for changing the health/disease patterns in the young population, the present study aims to identify schools with initiatives for promoting healthy eating and physical activity, and its relationship with the prevalence of overweight, hypertension, insulin resistance and hypercholesterolemia in adolescents in the city of Recife, Brazil.

## Methods

The study was conducted in the city of Recife (population, 1.538 million) in the state of Pernambuco, north-east Brazil. This work is part of the Study of Cardiovascular Risks in Adolescents *(ERICA - Estudo de Riscos Cardiovasculares em Adolescentes),* in the form of a cross-sectional, national, school-based study. Adolescents between 12 and 17 years of age without temporary or permanent physical disability who were enrolled in the last three grades/years of elementary school or in the three grades/years of high school in public and private schools in the city of Recife were included.

The sampling process tried to preserve the distribution of schools for administrative dependence, (public or private). In the sample, inside each geographical stratum, the method of systematic selection PPS (Proportional Probability to Size) was used, with a previous assortment of schools from the selection registry by geographical stratum, situation and administrative dependence. Thirty-nine (39) schools in Recife were selected in the sampling process. The sample is representative of the population of Recife, Brazil. A detailed description of the method can be found in Vasconcellos et al. [16].

In these schools, we evaluated the implementation of health promotion initiatives, estimated through interviews with the school manager and emphasizing the components described in Table 1.

**Table 1:** Description of the items evaluated at school

Components	Subcomponents	Evaluated items
Participation of school community	(annual) Meetings / Training	1) Average of meetings with parents to address the topic of Healthy eating 2) Average of meetings with employees to address the topic of Healthy eating 3) Training for teachers about Health and healthy eating
	Political-pedagogical project (PPP) construction/design	4) Suggestions from the meetings were considered for PPP construction/design 5) Involvement of School Community in the PPP construction/design 6) Involvement of teachers, directors and coordinators in PPP construction 7) Estimate of teachers involved in the PPP construction/design
	School curriculum	8) Inclusion of the Healthy eating thematic in the school curriculum 9) Inclusion of the health thematic in the school curriculum
		10) Inclusion of the Physical education thematic in the school curriculum
Presence of healthy environments	Healthy eating	1) Presence of a cafeteria with capacity to cover student demand during meals 2) Presence of specific space for disseminating information about health and healthy eating 3) Involvement of students in the construction of these spaces 4) These specific spaces are permanently at the school disposal 5) Presence of a space for cooking activities 6) Presence of a school vegetable garden to promote healthy eating 7) Presence of a commercial diner/cafeteria that promotes healthy eating or the absence of a commercial diner 8) Absence of vendors and/or a commercial store near the school
	Practicing physical activity	9) Presence of suitable environments for practicing physical activity 10) Practical course of physical activity routine during the week 11) Extra physical activity during the week
School nutrition policies		1) Policies to increase consumption of fresh foods 2) Policies to limit consumption of sweets, chips and soft drinks among students 3) Policies that dictate in which school events foods like fruits, whole grains should be included among the foods offered 4) Policies which indicate that food high in sugar, salt and fat and/or those with little nutritional value should not be included among the food offered at school 5) Policies that emphasize the processing degree of foods to students, such as ultra-processed foods (cookies, ice cream, soft drinks)
Monitoring of nutritional status		1) Monitoring the height and weight of students 2) Monitoring carried out for all students 3) Guidance to students who are overweight/obese developed by the school
Partnership with the health sector		1) Partnership/participation (voluntary or not) of a health care professional or in partnership with a clinic or hospital 2) Frequency of development of these actions/year (≥ 2 times/year) 3) Partnership with other institutions that promote Health and/or healthy eating

*PPP* Political-pedagogical project

Source: adapted from Silva et al. [15] and Roberts et al. [16]

The questionnaire composed by opened and closed questions was divided in four components: 1) participation of the school community (parents, teachers and students) in the school activities; 2) presence of healthy environments; 3) fellowship with the health sector; 4) monitoring of the nutritional state and 5) nutrition policy of the school. The items 1, 2, 3 and 4 were included based on the questionnaire of Silva et al. [15] However, component 4 was adapted for the present study to include the evaluation of the environment promoting the practice of physical activity. The component 5 and sub-component “Practicing physical activity” were elaborated based on the questionnaire The Health Behavior in School-aged Children-HBSC [17]. (Table 1).

A score system was used to ascertain the level of implementation of health promotion in the schools. Twenty-four (24) “yes or no” items received a positive score (1.0) for “yes”, or zero (0.0) for “no”. Eight (8) multiple-choice questions scored 0 when the action was not implemented, 1 for actions partially implemented, or 2 for actions implemented. The items referring to the “average number of meetings with the school community”, were considered positive when responses were higher than the average found in the sample. Missing data were considered null and marked as “0” in the analysis. The maximum sum of every item from each positively answered component was 40 points, distributed over 24 variables with a maximum score of one point each and 8 with a maximum score of 2.0.

By means of a simple rule of three, from the maximum score of the components (40 points) and the score obtained by each school, the level of implementation of each component was calculated, being defined as satisfactory when greater than or equal to 70% the implemented initiatives, unsatisfactory when below 70%. Joint analysis of these components allowed us to estimate the competency degree of initiatives for promoting healthy eating and the practice of physical activity developed by the schools. A detailed description of the components of school health promotion can be found in Bezerra et al. [18]

The weight of adolescents was obtained using a Líder® electronic scale with capacity of 200 kg and variation of 50 g. Height was measured using a portable and detachable Alturexata® stadiometer, with resolution of 1 mm and range of up to 213 cm. The specific procedures for each measurement are described in detail in Bloch et al. [19] BMI was calculated as body weight (kg) divided by the square of height (m). Reference curves were adopted according to the World Health Organization (WHO) [20] using the BMI-for-age index, according to gender. The adopted cut-off points were: Z-score ≥ − 2 and ≤1 (eutrophy); Z-score > 1 and ≤2 (overweight); Z-score > 2 (obesity). Adolescents with Z-score < − 3 (very low weight) or Z-score ≥ − 3 and < − 2 (low weight) were not included in the analyses.

Blood pressure was verified on the right arm using a digital monitor (Omron 705-IT) validated for use in adolescents, with the student sitting and following the recommendations in the literature [21]. Three consecutive measures were performed for each individual, where the first measure was discarded and the average of the last two measurements was used [19]. Adolescents were classified as: normotensive, if systolic and diastolic blood pressure were lower than the 95th percentile values for their height, gender and age; and as hypertensive, if the systolic or diastolic blood pressure corresponded to the 95th percentile or higher [21].

A single reference laboratory was used for blood collection, where all biochemical analyses of the study were centralized with strict quality control. Blood collection required 12-h fasting, and therefore it was only carried out on students in the morning classes/period who had been selected for the *ERICA* sample. Fasting glycemia was evaluated by GOD-PAP enzymatic method using Roche modular analytical equipment, fasting insulin by the chemiluminescence method, and cholesterol (mg/dL) by enzymatic kinetics method using Siemens ADVIA 2400 equipment. The HOMA-IR index (Homeostatic Model Assessment for Insulin Resistance) was calculated using fasting glucose and insulin values through the pre-established formula: HOMA-IR = (Fasting insulin x fasting glucose)/22.5. Cut-off points considered high were:

- Glucose 20: ≥ 126 mmol/l
- Fasting insulin 21: ≥ 20 mU/L
- Total cholesterol [22]: ≥ 170 mg/dL
- Insulin resistance 23 (HOMA-IR): ≥ 3.16

Prevalence and 95% confidence intervals (95% CI) were calculated from each component of the lipid, glycemic, overweight and hypertension profiles. Population estimates were obtained taking into account the complex sample design with weighting due to the different selection probabilities of the conglomerates (schools, grades/years and day-shift combinations, and classes) and later calibration by age and gender domains. The sample design can be found in Vasconcellos et al. [16].

Chi-square test and prevalence ratio (PR) with 95% CI were used as an association measure for analyzing the association between dependent variables and independent variables. Associations where *p* < 0.2 in the crude analysis were subjected to multivariate analysis to control any potential confounding factors. PRs and their 95% CI were calculated by Poisson regression analysis with robust variance adjustment in both crude and adjusted analysis. The level of statistical significance for the variables to remain in the final model was set at < 0.05. Analyses were carried out in the Stata statistical package 14.0 .

The study was approved by the Research Ethics Committees (CEP) of the Federal University of Pernambuco (Number 05185212.2.2002.5208) and the Aggeu Magalhães Research Center (CpqAM/Fiocruz) (Number 31446314.6.1001.5190). The adolescents who participated in this study agreed in writing to participate and their legal guardians signed an informed consent form.

## Results

A total of 2400 students participated in the study, of which 661 (27.54%) were from private schools and 1739 (72.46%) from public schools in the city of Recife. In this population, 1287 (53.63%) were males and 1113 (46.38%) were females.

The prevalence of overweight, obesity and hypertension among adolescents in the city of Recife was 16.88% (*n* = 389), 9.63% (*n* = 222) and 8.88% (*n* = 213), respectively. Biochemical parameters were only evaluated in the adolescents who studied in the morning (period), corresponding to 1126 evaluated students. The prevalence of hypercholesterolemia in this group was 20.62% (*n* = 232), while the prevalence of insulin resistance (HOMA-IR) was 11.75% (*n* = 132).

Table 2 presents the prevalence of overweight among adolescents, according to implementation of the components developed by the school to promote healthy eating and physical activity. The lower prevalence of overweight adolescents was found in schools that adequately implemented the “health promotion environments” component (*p* < 0.001). A higher prevalence of overweight/obesity was found in schools that did not have “partnerships with the health sector” (p < 0.001). Schools that were classified as “health promoters” had lower prevalence of overweight adolescents than schools where components were only “partially implemented” (*p* < 0.01).

**Table 2:** Prevalence of overweight among adolescents according to implementation of components for a health promoting school

Components	Population		Overweight		
	Obs	Estimated	(%)	PR (95% CI)	*p*
Participation of the school community					
Implemented ^a^	679	42.450	28.3	1	0.724
Unsatisfactory implementation ^b^	1.538	99.970	27.0	0.99 (0.95; 1.04)	
*(Annual) meetings/training sessions*					
Implemented ^a^	92	5.457	24.5	1	
Unsatisfactory implementation ^b^	2.125	136.963	27.5	1.01 (0.99; 1.04)	0.205
^d^ *PPP construction/design*					
Implemented ^a^	1968	130.242	27.5	1	
Unsatisfactory implementation ^b^	249	12.178	25.6	0.98 (0.96; 1.01)	0.413
*School curriculum*					
Implemented ^a^	1.696	111.070	28.4	1	
Unsatisfactory implementation ^b^	521	31.350	23.7	0.97 (0.94; 1.00)	0.092
Health promoting environment					
Implemented ^a^	141	8.322	20.8	1	< 0.001
Unsatisfactory implementation ^b^	2.076	134.098	27.7	1.04 (1.02; 1.06)	
*Healthy-eating promoting environment*					
Implemented ^a^	291	16.300	25.2	1	0.344
Unsatisfactory implementation ^b^	1.926	126.120	27.6	1.01(0.98; 1.04)	
*Promoting environment for physical activity*					
Implemented ^a^	297	20.197	30.0	1	0.492
Unsatisfactory implementation ^b^	1.920	122.223	27.0	0.98(0.93; 1.03)	
Nutrition policies					
Implemented ^a^	541	33.463	23.7	1	0.065
Unsatisfactory implementation ^b^	1.676	108.957	28.5	1.02 (0.99; 1.05)	
Monitoring of nutritional status					
Implemented ^a^	233	11.657	25.3	1	0.349
Unsatisfactory implementation ^b^	1.984	130.763	27.5	1.01 (0.98; 1.03)	
Partnership with the health sector					
Implemented ^a^	356	26.444	21.7	1	< 0.001
Unsatisfactory implementation ^b^	1.861	115.976	28.6	1.03 (1.01; 1.06)	
Health promoting school ^c^					
Implemented ^a^	196	12.214	22.4	1	0.001
Unsatisfactory implementation ^b^	2.091	130.206	27.8	1.01 (1.01; 1.04)	

^a^Implemented (70–100% of the developed initiatives)

^b^Unsatisfactory implementation: (< 69.9% of the initiatives developed)

^c^Joint analysis of all components

^d^*PPP* Political-pedagogical Project

The present study did not find statistical differences between hypertension and the components analyzed in schools, so only the abovementioned prevalence was described for this variable.

Table 3 shows that there is a statistically significant difference between the prevalence of adolescents with insulin resistance in schools that presented the “appropriate health promotion environment” component (7.7%), compared to those who presented an unsatisfactory implementation of this component (13.2%) (*p* < 0.05). Schools with adequate “monitoring of nutritional status” had lower prevalence of adolescents with insulin resistance than those who had unsatisfactory implementation (p < 0.05). The prevalence of insulin resistance in adolescents was greater among schools that did not present a partnership with the health sector or those that had an unsatisfactory partnership (*p* < 0.01).

**Table 3:** Prevalence of Insulin Resistance among adolescents according to implementation of components for health promoting school

Components	Population		Insulin Resistance		
	Obs	Estimated	(%)	PR (95% CI)	*p*
Participation of the school community					
Implemented ^a^	65	35.265	15.0	1	0.139
Unsatisfactory implementation ^b^	694	63.955	11.5	0.76 (0.53; 1.09)	
*(Annual) meetings/training sessions*					
Implemented ^a^	36	3.538	13.7	1	
Unsatisfactory implementation ^b^	1023	95.682	12.7	0.93 (0.75; 1.15)	0.469
^d^ *PPP construction/design*					
Implemented ^a^	947	89.353	13.4	1	
Unsatisfactory implementation ^b^	112	9.867	6.71	0.49 (0.34; 0.73)	0.001
*School curriculum*					
Implemented ^a^	916	86.075	12.3	1	
Unsatisfactory implementation ^b^	143	13.145	16.1	1.32(0.93; 1.85)	0.116
Health promoting environment					
Implemented ^a^	55	7.155	7.7	1	
Unsatisfactory implementation ^b^	1.004	92.065	13.2	1.71 (1.01; 2.88)	0.045
*Healthy-eating promoting environment*					
Implemented ^a^	127	13.217	8.2	1	0.005
Unsatisfactory implementation ^b^	932	86.003	13.5	1.63 (1.17; 2.27)	
*Promoting environment for physical activity*					
Implemented ^a^	158	19.136	13.8	1	0.553
Unsatisfactory implementation ^b^	901	80.084	12.5	0.9 (0.65; 1.96)	
Nutrition policies					
Implemented ^a^	186	23.173	11.0	1	0.416
Unsatisfactory implementation ^b^	873	76.047	13.3	1.2 (0.75; 1.96)	
Monitoring nutritional status					
Implemented ^a^	118	10.462	7.4	1	0.026
Unsatisfactory implementation ^b^	941	88.758	13.4	1.82 (1.08; 3.06)	
Partnership with the health sector					
Implemented ^a^	132	17.998	7.4	1	0.008
Unsatisfactory implementation ^b^	927	81.222	14.0	1.87 (1.19; 2.95)	
Health promoting school ^c^					
Implemented ^a^	84	11.694	7.8	1	0.062
Unsatisfactory implementation ^b^	975	87.526	13.4	1.72 (0.97; 3.04)	

^a^Implemented (70–100% of the developed initiatives)

^b^Unsatisfactory implementation: (< 69.9% of the initiatives developed)

^c^Joint analysis of all components

^d^*PPP* Political-pedagogical Project

Table 4 shows that in schools which included subjects related to health and healthy eating in the school curriculum, hypercholesterolemia prevalence in adolescents was 18.3%; while prevalence was 32.4% in schools where this subcomponent was not satisfactorily implemented, representing a statistically significant difference (p < 0.01). Prevalence of adolescents with hypercholesterolemia was also higher in schools that presented an unsatisfactory implementation of the component “healthy eating promoting environments”.

**Table 4:** Prevalence of hypercholesterolemia among adolescents according to implementation of components for a health promoting school

Components	Population		Hypercholesterolemia		
	Obs	Estimated	(%)	PR (95% CI)	*p*
Participation of the school community					
Implemented ^a^	365	35.240	18.2	1	0.371
Unsatisfactory implementation ^b^	696	63.980	21.2	1.17(0.82; 1.66)	
*(Annual) meetings/training sessions*					
Implemented ^a^	36	3.535	21.4	1	
Unsatisfactory implementation ^b^	1.025	95.685	20.1	0.93(0.77; 1.13)	0.497
^d^ *PPP construction/design*					
Implemented ^a^	949	89.363	20.6	1	
Unsatisfactory implementation ^b^	112	9.857	16.4	0.79(0.49; 1.28)	0.334
*School curriculum*					
Implemented ^a^	918	86.094	18.3	1	
Unsatisfactory implementation ^b^	143	13.126	32.4	1.76 (1.21; 2.60)	0.004
Health promoting environment					
Implemented ^a^	55	7.153	7.6	1	
Unsatisfactory implementation ^b^	1.006	92.067	21.1	2.77 (0.68; 11.21)	0.145
*Healthy-eating promoting environment*					
Implemented ^a^	127	13.203	15.8	1	0.006
Unsatisfactory implementation ^b^	934	86.017	20.8	1.32 (1.10; 1.60)	
*Promoting environment for physical activity*					
Implemented ^a^	158	19.116	14.5	1	0.118
Unsatisfactory implementation ^b^	903	80.104	21.5	1.48 (0.90; 2.44)	
Nutrition policies					
Implemented ^a^	186		18.4	1	0.588
Unsatisfactory implementation ^b^	875	76.062	20.7	1.12(0.72; 1.75)	
Monitoring of nutritional status					
Implemented ^a^	118	10.456	16.4	1	0.290
Unsatisfactory implementation ^b^	943	88.764	20.6	1.25(0.82; 1.91)	
Partnership with the health sector					
Implemented ^a^	132	17.990	18.0	1	0.615
Unsatisfactory implementation ^b^	929	81.230	20.6	1.15(0.65; 2.00)	
Health promoting school^d^					
Implemented ^a^	84	11.688	15.4	1	0.463
Unsatisfactory implementation ^b^	977	87.532	20.8	1.35 (0.58; 3.12)	

^a^Implemented (70–100% of the developed initiatives)

^b^Unsatisfactory implementation: (< 69.9% of the initiatives developed)

^c^Joint analysis of all components

^d^*PPP* Political-pedagogical Project

Variables that were submitted to multivariate analysis to test the independence of associations with overweight in adolescents were: “School curriculum”, “Nutrition policies”, “Health promoting environment” and “Partnership with the health sector”. After this procedure, schools where implementation of “Health promoting environment” (PR = 1.02; 95% CI: 1.0; 1.04) and of the “Partnership with the health sector” (PR = 1.03; 95%CI: 1.01; 1.05) were unsatisfactory remained significantly associated to overweight (Table 5).

**Table 5:** Adjusted prevalence ratio (PR) and its 95% confidence interval, according to selected variables for adolescents

	Components	PR (95% CI)	*p*
Overweight			
	Health promoting environment		
	Implemented ^a^	1	0.045**
	Unsatisfactory implementation ^b^	1.02 (1.0; 1.04)	
	Partnership with the health sector	1	
	Implemented ^a^	1.03 (1.01; 1.05)	0.001**
	Unsatisfactory implementation ^b^		
Insulin Resistance			
	Participation of the school community		
	Implemented ^a^	1	0.013**
	Unsatisfactory implementation ^b^	0.70 (0.53; 0.92)	
	Healthy-eating promoting environment		
	Implemented ^a^	1	0.024**
	Unsatisfactory implementation ^b^	1.36 (1.04; 1.79)	
	Partnership with the health sector		
	Implemented ^a^	1	0.008**
	Unsatisfactory implementation ^b^	2.12 (1.38; 3.24)	
Hypercholesterolemia			
	Curriculum includes Healthy eating and health topics		
	Implemented ^a^	1	
	Unsatisfactory implementation ^b^	1.71 (1.22; 2.44)	0.003**
	Healthy-eating promoting environment		
	Implemented ^a^	1	
	Unsatisfactory implementation ^b^	1.29(1.10; 1.54)	0.007**
	Physical activity promoting environment		
	Implemented ^a^	1	
	Unsatisfactory implementation ^b^	1.54 (0.95; 2.5)	0.074

* Prevalence Ratio (PR) adjusted for all variables included in the model. ** Indicates statistical significance (*p* < 0.05). N.B

^a^Implemented (70–100% of developed initiatives)

^b^Unsatisfactory implementation: (< 69.9% of developed initiatives)

The following variables were considered for analysis of Insulin Resistance: “Participation of the school community”, “PPP construction/design” (where PPP stands for political-pedagogical project, and it is equivalent to the school strategic project), “Healthy-eating promoting environment”, “Monitoring of nutritional status” and “Partnerships with the health sector”. Variables independently associated with Insulin Resistance were: unsatisfactory implementation of the “Participation of the school community” (PR = 0.70; 95% CI: 0.53; 0.92), “Healthy-eating promoting environment” (PR = 1.36; 95% CI: 1.04; 1.79), and “Partnership with the health sector” (PR = 2.12; 95% CI: 1.38; 3.24) (Table 5).

The variables independently associated with hypercholesterolemia were: unsatisfactory implementation of “Including themes on healthy eating and health in the school curriculum” (PR = 1.71; 95% CI: 1.22; 2.44) and “Healthy-eating promoting environment” (PR = 1.29; 95% CI: 1.10; 1.54). The other variables introduced in the adjusted model were not statistically significant (Table 5).

## Discussion

When properly implemented in the school environment, most of the evaluated initiatives for promoting healthy eating and physical activity showed a significant association with lower prevalence of overweight, insulin resistance and/or hypercholesterolemia, except for hypertension, which showed no significant association with the analyzed components.

In Brazil, we can consider that some events in the last two decades may have influenced schools to implement these initiatives, even if in an insipid way, for example: nutritional transition in children and adolescents; consequent policies of coping with these diseases; recognition of the school as a privileged space for health promotion; conducting national school health research; as well as strategies that aim at intersectoriality and involvement of basic health care with nutritional assessment actions and promoting healthy eating in schools.

Despite advances in diet, nutrition and physical activity policies in recent years, the prevalence of obesity found in Recife was high (9.6%), and higher than reported for the Northeast region (7.4%) in the same period [1]. The results of the study showed that 26.5% of adolescents are overweight; this is also a higher prevalence than what was described for adolescents living in Recife, Pernambuco, Brazil, in 2007 (20.4%) [23].

This situation is related to the fact that children and adolescents are growing in an obesogenic environment, caused by the energy imbalance resulting from changes in food types, availability, accessibility and commercialization, as well as a decline in physical activity due to more time spent in front of a screen and in sedentary leisure [4, 9, 24].

Overweight and obesity are the main contributors to the burden of chronic diseases in the population [2, 3]. The concern about the occurrence of obesity is related to developing comorbidities and complications generated by overweight. Fat accumulation is associated with the presence of arterial hypertension and metabolic alterations, such as the increase of triglycerides and glycemia, and reduced HDL-cholesterol [25], comorbidities of high prevalence in the present study.

These conditions are often asymptomatic at this stage of life, and can easily become unnoticed even by health professionals; therefore, emphasizing the importance of preventive and curative care in adolescents is crucial, as well as promoting healthy eating habits and to practice physical activity, which are among the main factors for the prevention of NCDs [26, 27]. In this sense, school is considered a facilitating space for interventions aimed at promoting healthy lifestyles, since it facilitates joint actions in the physical, social and educational environment [11]. At an individual level, health education through school curriculums is an important part of the school’s approach to health promotion [12].

National Curricular Parameters (*PCNs - Parâmetros Curriculares Nacionais)* guide promotion of health concepts across the school curriculum, as well as insertion of the “Healthy eating, physical activity and health” theme. An associated lower prevalence of hypercholesterolemia in adolescents was found in Recife when these themes were included in the school curriculum. In a study carried out in schools in São Paulo, the presence of themes related to health promotion was clear; however, other important issues are inconsistent such as access and purpose of diet in the processes of nutrition, eating habits, obesity and nutritional deficiencies [28].

These results demonstrate that schools that want to implement health promotion are often confronted with obstacles that they cannot overcome by themselves and therefore need properly support [29]. In Canada, public health units have played a key role in supporting this mandate through the development of curriculum resources, targeted classroom teaching related to promotion of health awareness campaigns and chronic disease prevention.

In Brazil the basic health units are developing these actions in the school environment [18]. However, it is understood that public health units may not have the capacity to offer support for all schools within their jurisdiction. In order to develop or maintain the capacity to facilitate and sustain healthy school communities, public health units may devise strategies for training public health and school staff, students and parents [30].

In the present study, school community participation is focused on involvement in meetings, constructing and executing the political pedagogical project, and training health educators, which may not have been comprehensive enough to assess the community’s actual participation in health and healthy eating promotion actions in school, and consequently their relationships with adolescents’ nutritional status. However, the literature recognizes that teachers, staff, and peers can encourage or even discourage physical activity behavior, and that this can influence adherence to weight control, improve social life and family relationships [31–33].

Davanço et al. [31] describe in their research that teachers exposed to the nutritional education course presented themselves better prepared conceptually and also more sensitized about the teacher’s and the school’s role as transformers of reality; demonstrating that the school environment can become a space for learning and for producing knowledge about health.

The presence of healthy environments in schools was associated with lower prevalence of overweight, IR (insulin resistance) and hypercholesterolemia in adolescents from Recife. These findings corroborate those by Dias et al. [9], who demonstrate that providing a supportive physical environment for healthy eating and for practicing physical activity should be considered in order to enable coping with chronic non-communicable diseases more effective.

Accessibility to sports fields and gyms, as well as the presence of equipment and a welcoming and safe environment promote physical activity in the school environment [32]. A systematic review [12] found that physical activity interventions in these conditions reduce body mass index (BMI) and that regular practice represents an important change in nutritional status in the school population.

In order to carry out actions that promote healthy eating within school, several environment possibilities can be considered, such as patios, snack shacks, vegetable gardens, cafeterias and classrooms. However, if used incorrectly, these environments can negatively influence students’ eating habit development. In evaluating the snacks offered in schools in Brasilia, Brazil, it was observed that they hardly used the cafeteria as a healthy eating promoting environment [13, 15]. These areas often offer ultra-processed, densely energetic, low-nutritional value foods [14].

In the present study, the presence of healthy eating environments in schools was associated with lower prevalence of IR, hypercholesterolemia. The presence of environments promoting healthy eating and physical activity was related to the lower prevalence of overweight in students. Howerton et al. [34] found that interventions in the school environment produced healthy lifestyle among students. That interventions were able to reduce students’ body mass index (BMI), increase physical activity and fitness levels, improve fruit and vegetable consumption [12].

Regarding nutrition policies for availability and encouragement of healthy eating in schools, no significant association was found with the overweight of adolescents. It is possible that this is due to limitations of the model used (cross-sectional study), as well as the implementation duration of these policies, considering that they need sufficient time to reflect into changes in eating habits and differences in BMI.

Similar findings were reported by Fernandes et al. [35], who found no significant differences in prevalence of overweight/obesity after the study due to an insufficient amount of time to notice changes in the nutritional status of the schoolchildren. Furthermore, schools may find it difficult to implement this component adequately, as they do not have partnerships with health professionals or even the support of government policies [18].

School meals do not currently appear to promote policies that encourage healthy eating among adolescents [18, 36]. School canteens sell or serve children and adolescents a daily diet of highly processed food, such as cookies with filling, bakery products, candy and soda. Consumption of such products early in life is directly related to high total cholesterol and LDL-cholesterol in children [37] and increased prevalence of metabolic syndrome in adolescents [38].

Some school policies have recently been effective in improving school meals and the school diet [39–41]. In Brazil, public schools have the support of the National School Meals Program (PNAE), which, despite its structural and financial limitations, provides school meals and dietary and nutritional education for students [42]. Fung et al. [43] evaluated quality before and after implementation of a school nutrition policy in the Canadian province of Nova Scotia and found that such interventions can have a positive impact on the quality of the diet, energy consumption and consumption of healthy beverages, but further action beyond the school setting is needed to reduce the prevalence of child obesity.

Schools with partnerships with the health sector presented a lower prevalence of adolescents with hypercholesterolemia and insulin resistance. This relationship is possibly a result of the fact that health professionals’ actions in schools can directly contribute to adolescents’ health; either directly, where the professional will develop actions along with the students; or considering an exchange of knowledge between health professionals and educators, this exchange can potentiate activities developed by teachers in the classroom with the students [15].

Despite the significant association in the crude analysis with lower prevalence of insulin resistance in adolescents, BMI lost significance in the multivariate analysis. Diagnosing BMI, and screening blood pressure, lipids and blood glucose are important measures to trace students’ needs to maximize health and to direct actions that will be developed with the school community. Moreover, it makes it possible to determine the magnitude, behavior and determinants of nutritional disorders, as well as to identify at risk groups and to direct appropriate interventions [2, 4, 10].

The lack of studies applying the same methodology to classify schools as health promoters has limited comparability of this study with others. Considering that this field is still under construction and few Brazilian studies have been conducted, it is necessary that further studies deepen the investigated components, to understand the school food environment, including the perception of what makes a health promoting school, and the barriers faced by teams trying to achieve this goal. Despite these limitations, these findings represent an important understanding of policies which influence the nutritional situation of schools/students.

## Conclusion

The results of the present study suggest that implementing components to promote healthy eating and physical activity, along with investments in health at schools, may contribute to reducing overweight, insulin resistance and hypercholesterolemia in adolescents, avoiding the onset of Chronic Non-communicable Diseases in adult life, thus contributing to developing healthy and productive adults, and consequently, avoiding suffering and reducing the inequality that these diseases can cause. Accordingly, they demonstrate the importance of healthy eating and practicing physical activity in school environments and its direct relationship with adolescents’ health. However, they also demonstrate that schools need more support to develop these activities, and there is a need for clear regulation that will help schools and their food services promote healthy eating in the school environment aimed at the well-being and health of students. Finally, it is necessary to mobilize the government and education and health departments to improve the physical spaces for practicing physical activity, and in designing, executing and monitoring these measures together with school managers, as well as adolescents’ nutritional status.

## Abbreviations

95% CI: 95% confidence intervals
BMI: Body Mass Index
PPP: Political-pedagogical project
PR: Prevalence Ratio
